# Cannabidiol modulation of hippocampal glutamate in early psychosis

**DOI:** 10.1177/02698811211001107

**Published:** 2021-04-16

**Authors:** Aisling O’Neill, Luciano Annibale, Grace Blest-Hopley, Robin Wilson, Vincent Giampietro, Sagnik Bhattacharyya

**Affiliations:** 1 Department of Psychosis Studies, King’s College London, London, UK; 2 Department of Psychiatry, Royal College of Surgeons in Ireland, Dublin, Ireland; 3 Department of Neuroimaging, King’s College London, London, UK

**Keywords:** Cannabidiol, psychosis, glutamate, proton magnetic resonance spectroscopy, hippocampus, schizophrenia

## Abstract

**Background:**

Emerging evidence supports the antipsychotic effect of cannabidiol, a non-intoxicating component of cannabis, in people with psychosis. Preclinical findings suggest that this antipsychotic effect may be related to cannabidiol modulating glutamatergic signalling in the brain.

**Aim:**

The purpose of this study was to investigate the effects of cannabidiol on the neurochemical mechanisms underlying psychosis.

**Methods:**

We investigated the effects of a single oral dose of cannabidiol (600 mg) in patients with psychosis, using a double-blind, randomised, placebo-controlled, repeated-measures, within-subject cross-over design. After drug administration, 13 patients were scanned using proton magnetic resonance spectroscopy to measure left hippocampal glutamate levels. Symptom severity was rated using the Positive and Negative Syndrome Scale 60 min before drug administration (pre-scan), and 270 min after drug administration (post-scan). Effects of cannabidiol on hippocampal glutamate levels, symptom severity, and correlations between hippocampal glutamate and symptoms were investigated.

**Results:**

Compared to placebo, there was a significant increase in hippocampal glutamate (*p*=0.035), and a significantly greater decrease in symptom severity (*p*=0.032) in the psychosis patients under cannabidiol treatment. There was also a significant negative relationship between post-treatment total Positive and Negative Syndrome Scale score and hippocampal glutamate (*p*=0.047), when baseline Positive and Negative Syndrome Scale score, treatment (cannabidiol vs placebo), and interaction between treatment and glutamate levels were controlled for.

**Conclusions:**

These findings may suggest a link between the increase in glutamate levels and concomitant decrease in symptom severity under cannabidiol treatment observed in psychosis patients. Furthermore, the findings provide novel insight into the potential neurochemical mechanisms underlying the antipsychotic effects of cannabidiol.

## Introduction

Δ9-Tetrahydrocannabinol (Δ9-THC), the primary psychoactive substance in cannabis, is a well-replicated risk factor for the onset and relapse of psychosis (Moore et al., 2007; Schoeler et al., 2016a, 2016b). Acute exposure has been shown to induce psychotic symptoms and alter activity in brain regions underlying psychotic symptoms in healthy individuals (Bhattacharyya et al., 2009, 2010; Bossong et al., 2012; D’Souza et al., 2004; Englund et al., 2013; Ranganathan et al., 2017; Schoeler and Bhattacharyya, 2013; Winton-Brown et al., 2011) and exacerbate symptoms in people with pre-existing psychosis (D’Souza et al., 2005). In contrast, cannabidiol (CBD), a non-intoxicating component of cannabis, has been shown to counteract these effects, displaying an antipsychotic effect as well as neural effects opposing that of Δ9-THC (Bhattacharyya et al., 2010, 2012a, 2015; Englund et al., 2013; Morgan et al., 2010; Winton-Brown et al., 2011). Further evidence regarding the antipsychotic efficacy of cannabidiol has emerged from independent clinical trials (Leweke et al., 2012; McGuire et al., 2017), though results have not always been consistent (Boggs et al., 2018). However, how CBD might exert its antipsychotic effect remains unclear. While current drugs for psychosis typically target the dopaminergic neurotransmitter system, it appears increasingly likely that CBD may directly or indirectly affect multiple distinct modes of neural signalling, including both glutamate and dopamine (Campos et al., 2016). Indeed, preclinical evidence suggests that CBD may attenuate the molecular changes induced by the antagonism of N-methyl-D-aspartate (NMDA) glutamate receptors (Gomes et al., 2014; Long et al., 2006; Moreira and Guimarães, 2005). This is consistent with evidence that CBD may increase glutamate levels in the ventromedial prefrontal cortex both acutely and following chronic treatment in freely moving animal models of depression, as well as acutely in healthy animals (Linge et al., 2016). However, an opposite effect on glutamate release, as measured in hippocampal synaptosomes, has also been reported in a cocaine-induced preclinical model of seizure (Gobira et al., 2015). A recent human study suggests that a single dose of CBD may increase Glx (the combination of glutamate and the glutamate metabolite glutamine) levels in the basal ganglia and decrease it in the dorsomedial prefrontal cortex in a group of healthy individuals (*n*=14) and individuals with autism spectrum disorder (*n*=9) (Pretzsch et al., 2019). However, whether CBD can modulate brain glutamate levels in patients with psychosis remains unclear.

Although a number of studies have investigated levels of glutamate and related metabolites as measured using proton magnetic resonance spectroscopy (^1^H-MRS) in patients with established psychosis, the results have been somewhat inconsistent (as demonstrated by three independent meta-analyses (Iwata et al., 2018; Marsman et al., 2013; Merritt et al., 2016). Indeed, there is evidence to suggest that these inconsistencies may be due to the stage of illness of the patient. Specifically, studies have reported levels of glutamate, glutamine, and/or Glx to be higher in the earlier stages of illness and lower in more chronic patients (Brandt et al., 2016; Duarte and Xin, 2019; Hashimoto et al., 2005; Ohrmann et al., 2005; Tayoshi et al., 2009; Tibbo et al., 2004). Some also suggesting a general decline with increasing age amongst patients but not controls (Brandt et al., 2016; Schwerk et al., 2014). This is also consistent with recent multimodal evidence suggesting an inverse relationship between cortical glutamate levels, and both striatal dopamine, and positive psychotic symptoms, in first episode psychosis patients (Jauhar et al., 2018).

We have recently shown that CBD may partially normalise activation in the prefrontal cortex (PFC) and medial temporal lobe (MTL) during a verbal memory task, as well as resting state functional connectivity between the hippocampus and striatum, in people in the early stages of psychosis (O’Neill et al., 2020). This is supported by our previous finding of a CBD-induced partial normalization of activation in the MTL and striatum during the same verbal memory task in people at clinical high-risk of psychosis (Bhattacharyya et al., 2018). However, to the best of our knowledge, no study has as yet investigated the effect of CBD on brain glutamate levels in patients with psychosis as a potential mechanism underlying its antipsychotic effects. Therefore, the main aim of this study was to investigate the acute effect of a single dose of CBD on left hippocampal glutamate levels and symptoms in people with established psychosis, using ^1^H-MRS. Previous research has demonstrated that the hippocampus is a key substrate for pathophysiological alterations in psychosis (Allen et al., 2016; Cao and Cannon, 2020; Lieberman et al., 2018; Lodge and Grace, 2011; McHugo et al., 2019; Schobel et al., 2013; Vargas et al., 2018). Independent evidence also suggests that cellular neuropathology may be evident in the left rather than right hippocampus in schizophrenia (Roeske et al., 2020). As such, previous magnetic resonance spectroscopy (MRS) studies have commonly acquired data from the left hippocampus (Bossong et al., 2019; Briend et al., 2020; Merritt et al., 2016; Shakory et al., 2018). Therefore, for the present study, we investigated hippocampal glutamate levels of the left hippocampus. We predicted that administration of a single dose of CBD would be associated with an increase in left hippocampal glutamate levels and reduction in symptoms of psychosis. A secondary objective was to explore the relationship between left hippocampal glutamate levels and CBD effect on symptoms.

## Methods

### Participants

Informed consent was obtained from all participants, as approved by the National Research Ethics Service Committee London (Camberwell, St Giles, Ethics reference: 14/LO/1861).

Patients with psychosis in the early stages of illness (within 5 years of onset) were recruited from psychiatric services in the South London and Maudsley National Health Service (NHS) Foundation Trust, in London, UK. Seventeen patients were initially recruited to the study, 15 of whom attended both study days – one patient did not meet the study inclusion criteria while another withdrew consent. Psychosis diagnosis was confirmed by an experienced research psychiatrist using the Structured Clinical Interview for the Diagnostic and Statistical Manual of Mental Disorders (DSM)-IV (American Psychiatric Association (APA), 2000). Briefly, inclusion criteria were as follows: (a) diagnosis of psychotic mental illness (meeting criteria for schizophrenia, schizophreniform or brief psychotic disorder – but no other Axis I diagnoses) and (b) within 5 years of onset of illness. Additional inclusion criteria as well as exclusion criteria are described in the Supplementary Material section.

### Study design

Patients were studied over two sessions in a double-blind, placebo-controlled, repeated-measures, within-subject-crossover design, with at least a one-week interval (mean=28.87 days, range=6–114 days) between scans to allow washout of CBD (>3 times the elimination half-life of CBD (Devinsky et al., 2014; Hawksworth and McArdle, 2004)). A randomization sequence was generated by a research pharmacist at the South London and Maudsley NHS foundation Trust, and was used by the pharmacist to allocate patients to one of the two orders of drug administration (placebo followed by CBD or CBD followed by placebo).

On each study day, patients had a light standardised breakfast. Patients were given a gelatin capsule containing either CBD 600 mg (approx. 99.9% pure, Δ9-THC-Pharm, Frankfurt, Germany), or a visually identical placebo (PLB) capsule containing flour 120 min after breakfast. Participants then underwent ^1^H-MRS scanning 180 min after drug administration. Blood samples were obtained via intravenous cannulation in the non-dominant arm to assay CBD levels at three time points: 60 min before drug administration (T1), 60 min after drug administration (T2), and 270 min after administration (T3) (see Supplementary Material).

Psychopathology was rated in patients using the Positive and Negative Syndrome Scale (PANSS; Kay et al., 1987), administered 60 min before drug administration (T1; pre ^1^H-MRS scan), and again 270 min after administration (T3; post ^1^H-MRS scan). ^1^H-MRS spectra were acquired 180 min after drug administration.

All participants were advised to avoid alcohol intake for 24 h and caffeine intake for 12 h before the study. Additionally, all participants were asked to avoid using any recreational drugs (apart from cannabis amongst the patient group) for two weeks before the study day. A urine sample was obtained from all participants on each study day to screen for use of amphetamines, barbiturates, benzodiazepines, cocaine, methamphetamine, morphine, methadone, phencyclidine, tricyclic antidepressants and Δ9-THC, using the Alere Drug Screen Urine Test Cup. Carbon monoxide breath levels were also measured in the patient group, using the Bedfont Smokerlyzer.

### ^1^H-MRS data acquisition

^1^H-MRS resting-state spectra (Point RESolved Spectroscopy (PRESS); echo time (TE)=30 ms; repetition time (TR)=3 s; 96 averages, additional details in Supplementary Material) were acquired in the left hippocampus, as previously described by Stone et al. (2010), on a GE SIGNA HDx 3.0T MR scanner system (GE Medical Systems, Milwaukee, Wisconsin). We employed the standard GE probe (proton brain examination) sequence, using a standardised chemically selective suppression (CHESS) water suppression routine. For each metabolite spectrum, unsuppressed water reference spectra (16 averages) were also acquired as part of the standard acquisition. Shimming and water suppression were optimised, with auto-prescan performed twice before each scan. Using standardised protocols, the hippocampal region of interest (20×20×15 mm; right-left, anterior-posterior, superior-inferior) was prescribed from the structural T1 scan (see Supplementary Material). T1 images were acquired using a whole-brain 3D sagittal ADNI GO sequence, with the following parameters: TR/TE=6.98/2.85 ms; flip angle=11°; 196×1.2 mm slices; voxel size 1.0×1.0×1.2 mm; 256×256 matrix.

### ^1^H-MRS quantification

All spectra were analysed with LCModel version 6.3-1L (see example spectra in the Supplementary Material) (Provencher, 1993). Cramer-Rao minimum variance bounds (as reported by LCModel) were less than 20% for all peaks, thus no subjects were excluded from the analyses following quality control checks. Water-scaled glutamate (Glu) values were corrected for cerebral spinal fluid (CSF) content of the hippocampal region of interest (ROI) using the formula

$$Mcorr=M^{*}\left(WM+1.21GM+1.55CSF\right)/\left(WM+GM\right)$$

where M is the uncorrected metabolite value, and WM, GM and CSF are the white matter, grey matter and CSF fractions of the ROI, respectively (Egerton et al., 2014). The WM, GM and CSF fractions were determined for each subject from the structural T1 scans, and subsequently segmented into GM, WM and CSF using SPM8 (https://www.fil.ion.ucl.ac.uk/spm/software/spm8/). As exploratory measures, CSF-corrected water-scaled values for glycerophosphocholine (GPC), combined glutamate and glutamine (Glx), N-acetyl aspartate (NAA), and myoinositol (mI) were also collected. All CSF-corrected metabolite values are reported in arbitrary units. ^1^H-MRS data quality is described in Supplementary Material.

### Additional analyses

Effect of CBD treatment on hippocampal Glu levels and symptoms was assessed using the non-parametric repeated measures Sign test. The Sign test was used as it has fewer assumptions (appropriate given the modest sample size) than standard parametric tests and is thus more conservative. Linear regression analysis was carried out to examine the relationship between severity of symptoms of psychosis following treatment (as indexed by total PANSS score at T3) and hippocampal Glu concentration after controlling for the effect of baseline symptoms (total PANSS score at T1), treatment (CBD vs PLB) and interaction between treatment and hippocampal Glu. Exploratory analyses of the effect of CBD treatment on the additional metabolites were also performed using the non-parametric repeated measures Sign test. All analyses were carried out using the R Stats package (R Core Team, 2018).

## Results

### Demographics and behavioural findings

Socio-demographic and clinical data are shown in Table 1 (additional details, including cannabis use, in Supplementary Material). Symptom ratings at pre-treatment (T1), and change following drug administration, on each of the study days are shown in Table 2.

**Table 1.** Participant socio-demographic information.

Characteristic	PSY (*n*=15)
	Mean (SD)
Age in years	27.73 (4.61)
Gender (% male)	66.7
Handedness (% right)	86.7
Education in years	14.23 (2.75)
Antipsychotic medication (atypical/typical/none)	14/0/1^a^
CPZ equivalent dose mg/day	225.071 (96.22)

PSY: psychotic; SD: standard deviation, CPZ: clozapine.

a Patient was prescribed olanzapine, but was not taking it.

**Table 2.** Symptom scores for psychosis (PSY) patients at baseline (T1) and post-drug (T3) for both study days.

Characteristic	PSY-PLB	PSY-CBD	*Statistics*
	Mean (SD)		
T1 PANSS positive symptoms	12.53 (5.62)	12.93 (5.72)	Z=0, *p*-value=0.5
T1 PANSS negative symptoms	12.4 (6.4)	12.47 (6.56)	Z=−0.28, *p*-value=0.39
T1 PANSS general symptoms	23.87 (8.6)	25.6 (8.83)	Z=−1.29, *p*-value=0.098
T1 PANSS total symptoms	48.8 (18.9)	51 (20)	Z=−1.069, *p*-value=0.14
T3 PANSS positive symptoms	11.67 (4.99)	10.73 (3.41)	Z=0.301, *p*-value=0.62
T3 PANSS negative symptoms	11.53 (6.058)	10.2 (3.052)	Z=1.15, *p*-value=0.87
T3 PANSS general symptoms	21.4 (8.1)	20.6 (6.16)	Z=1.39, *p*-value=0.92
T3 PANSS total symptoms	44.6 (18.07)	41.53 (11)	Z=0.904, *p*-value=0.82
Change PANSS positive symptoms (T1–T3)	0.87 (2.2)	2.2 (4.11)	Z=−0.83, *p*-value=0.2
Change PANSS negative symptoms (T1–T3)	0.87 (2.82)	2.27 (4.17)	Z=−1.508, *p*-value=0.066
Change PANSS general symptoms (T1–T3)	2.47 (3.46)	5 (6.45)	Z=−1.6, *p*-value=0.054
Change PANSS total symptoms (T1–T3)	4.2 (7.61)	9.47 (14.17)	Z=−2.14, *p*-value=0.016

PANSS: Positive And Negative Syndrome Scale.

PSY-CBD refers to patients under cannabidiol condition; PSY-PLB refers to patients under placebo condition; T1 is the time-point at 60 min before drug administration; T3 is the time-point at 270 min after drug administration. Significant differences are indicated in bold.

On the PLB treatment day, one patient was missing five T1 general PANSS item scores; one patient was missing one T3 positive PANSS score; and one patient was missing two T3 negative PANSS scores. On the CBD treatment day, one patient was missing seven T3 negative PANSS item scores. In these cases, the last observation carried forward method was used to impute the data – wherein a participant’s last observed score on the dependent variable is used for all subsequent (i.e. missing) observation points.

**Figure 1.**
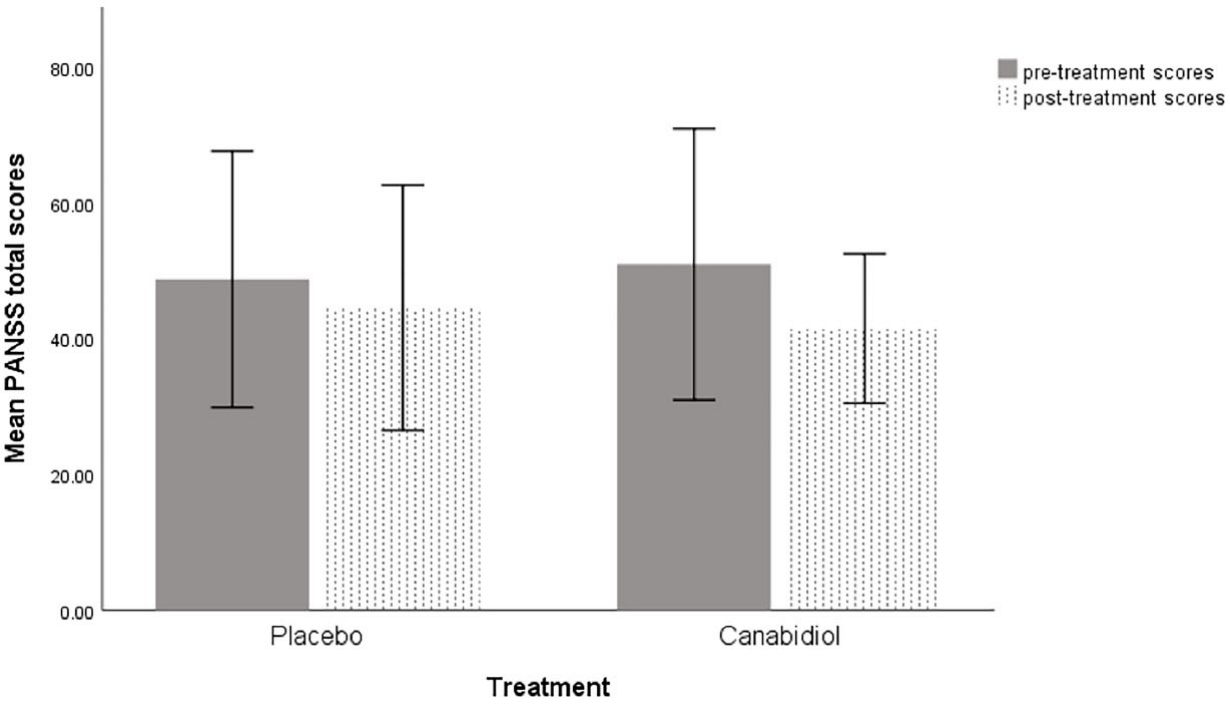
Change in total Positive and Negative Syndrome Scale (PANSS) scores pre- and post-treatment on the placebo and cannabidiol treatment days. Error bars=±1 standard deviation (SD).

Symptoms at baseline (T1, pre-treatment) on each study day, as indexed by PANSS total scores were not significantly different between the CBD (PSY-CBD) and PLB (PSY-PLB) conditions (*z*=−1.069, one-tailed *p*=0.14). Across both treatment conditions, severity of psychosis symptoms as indexed by PANSS total scores improved following drug treatment, compared to baseline. This improvement was significantly greater under the CBD treatment condition than under the PLB treatment condition (*z*=−2.14; *p*=0.016) (Table 2, Figure 1).

### Differences in glutamate levels across the groups

**Figure 2.**
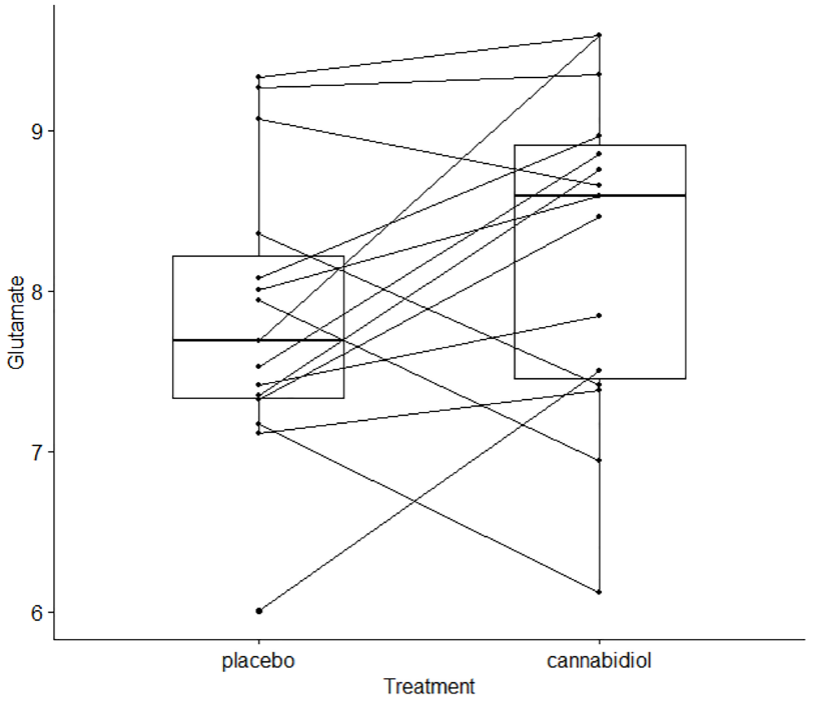
Cannabidiol (CBD)-related difference in left hippocampal glutamate levels, within individuals with psychosis. Hippocampal glutamate is presented as data corrected for voxel cerebrospinal fluid content, in arbitrary units (AU).

As predicted, there was a significant increase in left hippocampal glutamate levels under the CBD treatment condition (8.27±1.023) compared to the PLB treatment condition (7.84±0.9) (*z*=−1.80; one-tailed *p*=0.035) (Table 3, Figure 2).

**Table 3.** Metabolite levels for psychosis (PSY) patients under placebo (PLB)/cannabidiol (CBD) conditions.

Metabolite	PSY-PLB	PSY-CBD	Statistics	Cohen’s *d*
	Mean (SD)			
Glutamate	7.84 (0.9)	8.27 (1.023)	**PSY-PLB vs PSY-CBD: *p***=**0.035**	PSY-PLB vs PSY-CBD: 0.48
Glutamate+glutamine	11 (1.92)	11.46 (1.19)	PSY-PLB vs PSY-CBD: *p*=0.79	PSY-PLB vs PSY-CBD: 0.24
Myoinositol	6.27 (1.064)	6.41 (1.11)	PSY-PLB vs PSY-CBD: *p*=0.79	PSY-PLB vs PSY-CBD: 0.13
N-acetyl aspartate	9.17 (1.18)	9.37 (1.26)	PSY-PLB vs PSY-CBD: *p*=0.79	PSY-PLB vs PSY-CBD: 0.17
Glycerophosphocholine	2.41 (0.28)	2.37 (0.29)	PSY-PLB vs PSY-CBD: *p*=0.59	PSY-PLB vs PSY-CBD: 0.14

SD: standard deviation.

PSY-CBD refers to patients under cannabidiol condition; PSY-PLB: refers to patients under placebo condition. Significant differences are indicated in bold.

### Differences in additional metabolite levels across the groups

No significant differences were observed between the PLB and CBD conditions, for any of the additional metabolites (Table 3).

### Relationship between hippocampal glutamate and psychosis symptoms

**Figure 3.**
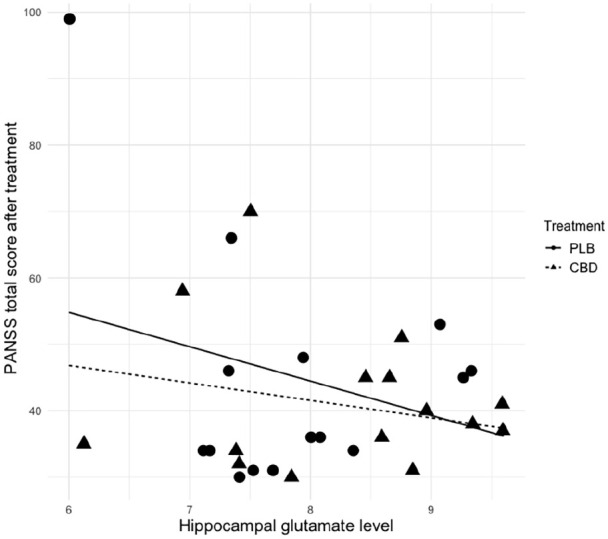
Significant negative relationship between hippocampal glutamate and Positive and Negative Syndrome Scale (PANSS) total score post treatment (*p*=0.047), controlling for the effect of baseline symptoms (total PANSS score at T1), treatment (cannabidiol (CBD) vs placebo (PLB)), and interaction between treatment and hippocampal glutamate levels.

There was a significant negative relationship between post-treatment (T3) total PANSS score and hippocampal Glu concentration (*p*=0.047), even after controlling for the effect of baseline symptoms (total PANSS score at T1), treatment (CBD vs PLB), and interaction between treatment and hippocampal Glu levels (Table 4, Figure 3).

**Table 4.** Regression model showing the effect of hippocampal glutamate (Glu) on total Positive and Negative Syndrome Scale (PANSS) score post-treatment.

Predictor	Estimate	Std error	*t* value	*p* value
T1 PANSS total symptoms	0.61	20.37	2.709	**<0.001**
Group (CBD)	−23.26	26.75	−0.87	0.39
Hippocampal Glu	−5.17	2.48	−2.083	**0.047**
Group (CBD): Hippocampal Glu	2.54	3.31	0.77	0.45

CBD: cannabidiol.

Results of the linear regression analysis examining the relationship between severity of total PANSS symptoms following treatment (as indexed by total PANSS score at T3) and hippocampal Glu concentration, after controlling for the effect of baseline symptoms (total PANSS score at T1), treatment (CBD vs placebo), and interaction between treatment and hippocampal Glu. Adjusted *R*^2^=0.68. Significant differences are indicated in bold.

## Discussion

In this study, as predicted, a single dose of CBD given to patients with psychosis significantly increased hippocampal glutamate levels. This was accompanied by a significantly greater reduction in symptoms of psychosis, as indexed by total PANSS score, under CBD compared to PLB treatment. Furthermore, there was a significant inverse relationship between hippocampal glutamate levels and severity of psychosis symptoms post-treatment, even after controlling for baseline symptom severity and treatment. However, this relationship did not seem to be significantly different under CBD compared to PLB treatment.

Overall, these findings may suggest an inverse relationship between hippocampal glutamate levels and symptoms of psychosis. As there was an increase in glutamate levels and a concomitant acute decrease in symptom severity under CBD, this may also suggest that the acute improvement in symptoms of psychosis under CBD may be linked to the increase in hippocampal glutamate concentration, as indexed by ^1^H-MRS, under its influence. However, this should be interpreted with caution, as the correlation between hippocampal glutamate and symptoms was not selective for CBD exposure. Previous studies linking glutamate levels to symptoms in patients with psychotic disorder have been inconsistent, as reviewed by Merritt et al. (2013). However, administration of NMDA receptor antagonists has been shown to induce the full spectrum of psychotic symptoms in healthy individuals (Javitt, 2007; Newcomer et al., 1999), and worsen positive, negative and cognitive symptoms in patients with psychosis (Lahti et al., 1995; Malhotra et al., 1997). The direction of the CBD-induced change in glutamate level observed in the current study is in keeping with recent clinical and preclinical evidence (Linge et al., 2016; Pretzsch et al., 2019). It is also consistent with previous studies reporting lower levels of glutamate in more chronic compared to un-medicated patients or those in earlier stages of illness (Schwerk et al., 2014).

As noted earlier, traditional drugs for psychosis typically target the dopaminergic neurotransmitter system, varying in efficacy in the treatment of positive symptoms, and providing only modest benefit in the treatment of the negative and cognitive symptoms of psychosis (Beck et al., 2016). In contrast, glutamatergic models of psychosis may provide more inclusive explanations for the psychotic symptoms and cognitive impairments observed in the disorder, with hypofunction of the NMDA receptors being a prominent pathophysiological feature (Coyle, 1996; Li and Tsien, 2009; Newcomer et al., 1999). Such models are supported by the psychotomimetic effects of NMDA receptor antagonists in healthy individuals (Javitt, 2007; Newcomer et al., 1999); and by the discovery of abnormal markers of NMDA receptor neurotransmission in post-mortem brains (Beneyto et al., 2007; Kristiansen et al., 2006), and abnormal levels of glutamatergic metabolites throughout the brain *in vivo* (Bartha et al., 1997; Bustillo et al., 2017; Tayoshi et al., 2009), in patients with psychosis. The ubiquitous distribution of glutamate receptors throughout the brain, and the crucial role of NMDA receptors specifically in the modulation of synaptic plasticity, further implicate the disruption of these receptors in widespread dysfunction (Howes et al., 2015; Li and Tsien, 2009). However, clinical trials of glutamatergic drugs for psychosis have been largely unsuccessful (Beck et al., 2016). Although the current findings are promising in terms of suggesting a potential glutamatergic mechanism for candidate drugs for psychosis, previous experience with glutamatergic treatments indicate that there is a particular need for caution, pending independent replication. Further studies involving sustained treatment with CBD are also needed, particularly exploring the relationship between change in pre-treatment glutamate levels and the effect of CBD on symptoms as well as cognitive impairments in psychosis.

The molecular mechanisms that may underlie the effect of CBD on glutamate levels in patients are unclear as are the molecular mechanisms that may underlie its antipsychotic effects. A range of mechanisms have been suggested as potentially operative either individually or in conjunction with others to produce the antipsychotic and anxiolytic effects of CBD (Leweke et al., 2012; Mechoulam et al., 2007). Some of these include the inhibition of adenosine reuptake (Carrier et al., 2006), agonism of the transient potential vanilloid-1 (TRPV-1) and serotonergic 5-HT1A receptors (Bisogno et al., 2001; Russo et al., 2005), and partial agonism of the dopamine D2High receptor (Seeman, 2016). Agonism of the pre-synaptic TRPV-1 receptor in particular can also facilitate glutamate release (Xing and Li, 2007). Another prominent potential mechanism involves the endocannabinoid system. It is thought to play a critical role in regulating glutamatergic signalling, modulating NMDA receptor activity in order to prevent excitotoxicity (Sanchez-Blazquez et al., 2014). The endocannabinoid system has also been strongly implicated in the pathophysiology of psychosis in both clinical and pre-clinical studies (Appiah-Kusi et al., 2016; Bhattacharyya et al., 2012b; Bossong et al., 2014; Dalton et al., 2011; D’Souza et al., 2004; Leweke et al., 2016; Muguruza et al., 2013). It follows that aberrant activity of the endocannabinoid system resulting in NMDA receptor hypofunction could support the glutamatergic model of psychosis (Sanchez-Blazquez et al., 2014). Furthermore, CBD has been shown to disrupt the action of agonists of the cannabinoid 1 (CB1) receptor, the primary endocannabinoid receptor. In this way, CBD-induced modulation of hippocampal glutamate levels and concomitant improvement in psychotic symptoms may suggest that an effect on hippocampal endocannabinoid tone could underlie the antipsychotic effects of CBD. Whatever the mechanism is that ultimately underlies the effect of CBD on hippocampal glutamate, it is important to note that this process and outcome may not reflect the effect in other brain regions, given the findings of previous clinical and pre-clinical studies (Gobira et al., 2015; Linge et al., 2016; Pretzsch et al., 2019), as well as their relationship with symptomatic effects.

The main limitation of this study was its relatively modest sample size. We attempted to mitigate this by employing a within-subject repeated measures design, allowing each participant to act as their own control. This helped minimise the effect of between-subject differences such as concomitant antipsychotic medications and comorbid drug use. Nevertheless, we cannot completely rule out that some of the changes in hippocampal glutamate observed in the present study were related to concomitant treatment with antipsychotic medication. However, further studies are needed to confirm these findings in larger samples of patients, and to examine whether these effects persist after a sustained period of treatment. Another limitation worth considering relates to the potential confounding of the current results by the carry-over effects of CBD in participants who received it on their first study session. While we aimed to limit this by using a counter-balanced order of administration, ultimately, we had 15 patients who completed the study, with seven receiving PLB first, and eight receiving CBD first. However, our between-session interval was substantially over the typical recommended threshold of three times the elimination half-life of CBD (Devinsky et al., 2014), making it unlikely that any carry-over effects of CBD would have persisted by the second study session. Furthermore, post-hoc analyses revealed no significant differences in change in glutamate levels (CBD minus PLB condition) between those who received PLB first, compared to those who received CBD first (data not shown here). Nevertheless, we cannot completely rule out this possibility.

In conclusion, we identified that a single dose of CBD modulated hippocampal glutamate levels and symptoms of psychosis, in patients with established psychosis. These findings are in keeping with the purported antipsychotic effects of CBD in psychosis, and provide novel insight into potential neurochemical mechanisms underlying these effects.

## Supplemental Material

sj-docx-1-jop-10.1177_02698811211001107 – Supplemental material for Cannabidiol modulation of hippocampal glutamate in early psychosis Supplemental material, sj-docx-1-jop-10.1177_02698811211001107 for Cannabidiol modulation of hippocampal glutamate in early psychosis by Aisling O’Neill, Luciano Annibale, Grace Blest-Hopley, Robin Wilson, Vincent Giampietro and Sagnik Bhattacharyya in Journal of Psychopharmacology
