# Supplementary material for: Cannabidiol modulation of hippocampal glutamate in early psychosis
Source: J Psychopharmacol. 2021 Apr 16;35(7):814–22. doi: 10.1177/02698811211001107 (PMC8278563; doi:10.1177/02698811211001107)
Supplement: sj-docx-1-jop-10.1177_02698811211001107 – Supplemental material for Cannabidiol modulation of hippocampal glutamate in early psychosis [file sj-docx-1-jop-10.1177_02698811211001107.docx]

# Effect of Cannabidiol on functional activation and hippocampal glutamate in psychosis: a multimodal analysis

Aisling O'Neill^1^, Luciano Annibale^1^, Grace Blest-Hopley^1^, Robin Wilson^1^, Vincent Giampietro^2^, Sagnik Bhattacharyya^1^*

# Methods

**Full inclusion criteria for patients with psychosis**

Inclusion criteria were as follows: (1) diagnosis of psychotic mental illness (meeting criteria for schizophrenia, schizophreniform, or brief psychotic disorder – but no other Axis I diagnoses), (2) within 5 years of onset of illness, (3) receiving a stable dose of antipsychotic medication for ≥3weeks and stable enough to comply with neuroimaging, (4) engaged with early-intervention services.

**Exclusion criteria**

Exclusion were as follows: (1) a history of neurological disorder (e.g. epilepsy), severe inter-current illness or pregnancy, (2) alcohol and other current substance dependence (excluding cannabis), (3) acute intoxication with any other psychoactive substance on the day of experimentation, (4) an IQ of less than 70 or lack of capacity to consent, (5) urine drug screen positive for other known psychotogenic and psychedelic substances such as PCP, amphetamine or MDMA (past use that does not result in a positive urine drug screen result on study days and also does not satisfy criteria for dependence did not result in exclusion), (6) any contraindications to MRI scanning.

**Study Design**

Blood samples were subjected to centrifugation for 10 minutes/3000rpm, at 4°C, and stored at -80°C for further processing. Plasma concentrations of CBD were measured by the Mass Spectrometry Facility, King's College London.

The 600mg dose of CBD, and subsequent absorption period (180 min between drug administration and fMR imaging), were chosen based on the findings of previous studies generally describing CBD symptom effects and plasma levels peaking 3 hours following oral administration (Agurell et al., 1981, Bergamaschi et al., 2011, Bhattacharyya et al., 2010, Fusar-Poli et al., 2010, Martin-Santos et al., 2012).

**1H-MRS quantification**

The low bandwidth (around 2 kHz for the excitation pulse and 1 kHz for the refocusing pulses) of the PRESS localisation pulses mean that the PRESS localisation suffers from chemical shift displacement error. This leads to a displacement of around 2.5 mm/parts per million (ppm) in the directions defined by the refocusing pulses and around 1.3 mm/ppm for the excitation pulse (for a 2 cm voxel). We mitigated this with two approaches. Firstly, we set the transmit frequency for the localisation pulses to 2.7 ppm, roughly half-way between the centre of the creatine & choline peaks and the NAA peak. As a result, peaks at lower and higher frequencies than the transmit frequency are shifted in different directions, thereby minimising the largest displacement compared to that we would have seen if we had set the transmit frequency to that of water. Secondly, we use Outer Volume Saturation (OVS) with Very Selective Saturation (VSS) pulses. As they have a high bandwidth (11 kHz), they do not suffer from as much chemical shift displacement error as the PRESS localisation pulses (around 0.2 mm/ppm). The OVS bands are placed adjacent to the PRESS localised voxel and suppress signal from outside the PRESS localised voxel. The OVS also have a very sharp cut-off between minimum and maximum saturation, unlike the PRESS localisation pulses (especially the refocusing pulses) which have a more gradual decline in spatial profile. This ensures that the voxel cut-off is sharper than using PRESS localisation alone and reduces the effect of the pulse profiles of the PRESS localisation pulses.


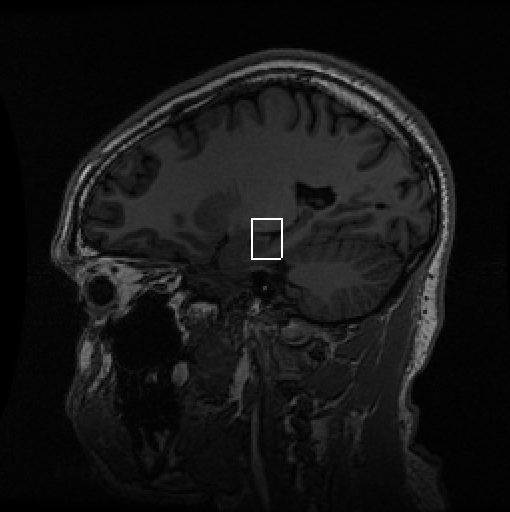


Figure S1: Sagittal image example of magnetic resonance spectroscopy (MRS) voxel placement in the left hippocampus.


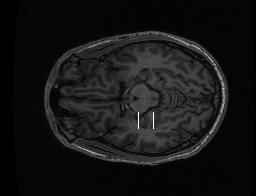


Figure S2: Axial image example of magnetic resonance spectroscopy (MRS) voxel placement in the left hippocampus.

# Results

| **Characteristic** | **Psychosis patients (n = 15)** | |
| --- | --- | --- |
|  | **Mean (SD)** | |
| **Number of hospital admissions** | 1.54 (0.97) | |
| **Urine Drug screen results:** | *Visit 1* | *Visit 2* |
|  | 6 | 6 |
| Δ9-THC | 8 | 8 |
| Morphine | 0 | 0 |
| Benzodiazepines | 0 | 0 |
| PCP* | 1 | 1 |
| **Lifetime cannabis use:** | 15 exposed (9 current regular users) | |
| **Frequency of cannabis use (past/present):** | | |
| Daily | 6 | |
| More than once a week | 4 | |
| Once/twice monthly | 0 | |
| Few times a year | 1 | |
| Only once/twice lifetime | 4 | |
| **Lifetime alcohol use:** | 11 exposed (7 current users) | |
| **Frequency of alcohol use (past/present):** | | |
| Daily | 1 | |
| More than once a week | 3 | |
| Once/twice monthly | 3 | |
| Few times a year | 3 | |
| Never | 4 | |
| Missing | 1 | |
| **Lifetime nicotine use:** | 7 exposed (6 current users) | |
| Frequency of nicotine use (past/present): | | |
| Daily | 6 | |
| More than once a week | 0 | |
| Once/twice monthly | 1 | |
| Few times a year | 0 | |
| Never | 8 | |
| Missing | 0 | |

*one patient tested positive for phencyclidine (PCP) on both occasions. However, this finding was disregarded as the patient was receiving concurrent venlafaxine treatment, known to cause false positive results for PCP (Sena et al., 2002, Santos et al., 2007). Δ9-THC = Δ-9-tetrahydrocannabinol.

*
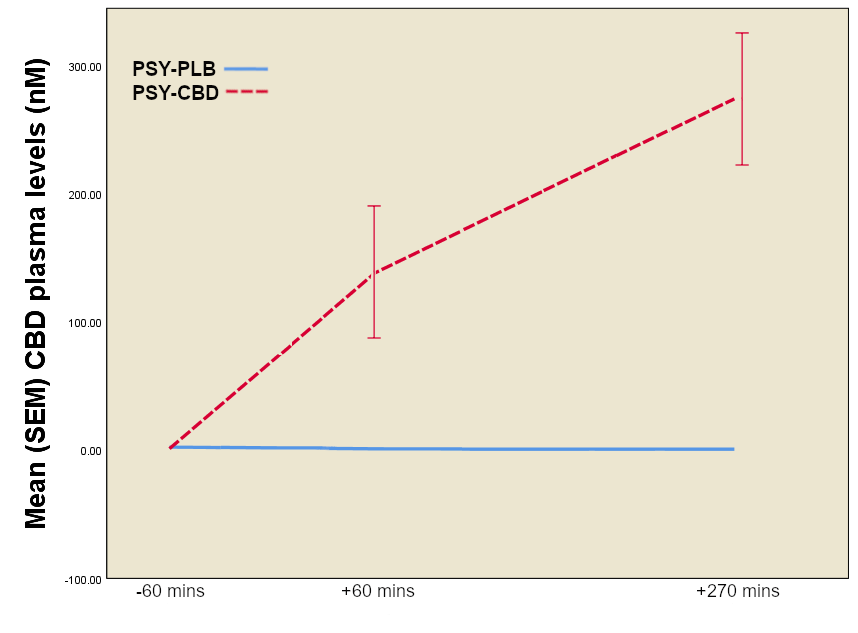
*

Figure S2: Plot showing CBD plasma levels in the psychosis patients under placebo (PSY-PLB) or CBD (PSY-CBD) condition, at 60 min before drug administration (-60 min), 60 min after drug administration (+60 min), and 270 min after drug administration (+270 min). Error bars: +/- SE.

**Missing PANSS and STAI-S data**

On the placebo treatment day, one patient was missing 5 T1 general PANSS item scores; one patient was missing 1 T3 positive PANSS score; and one patient was missing 2 T3 negative PANSS scores. Also on the placebo treatment day; one patient was missing 3 T1 STAI-S item scores; two patients were missing 1 T1 STAI-S item scores, each; and another two patients were missing 1 T3 STAI-S item scores, each. On the CBD treatment day, one patient was missing 7 T3 negative PANSS item scores. Also on the CBD treatment day, two patients were missing 1 T1 STAI-S item scores, each; and four patients were missing 1 T3 STAI-S item score, each. In these cases, the Last Observation Carried Forward method was used to impute the data.


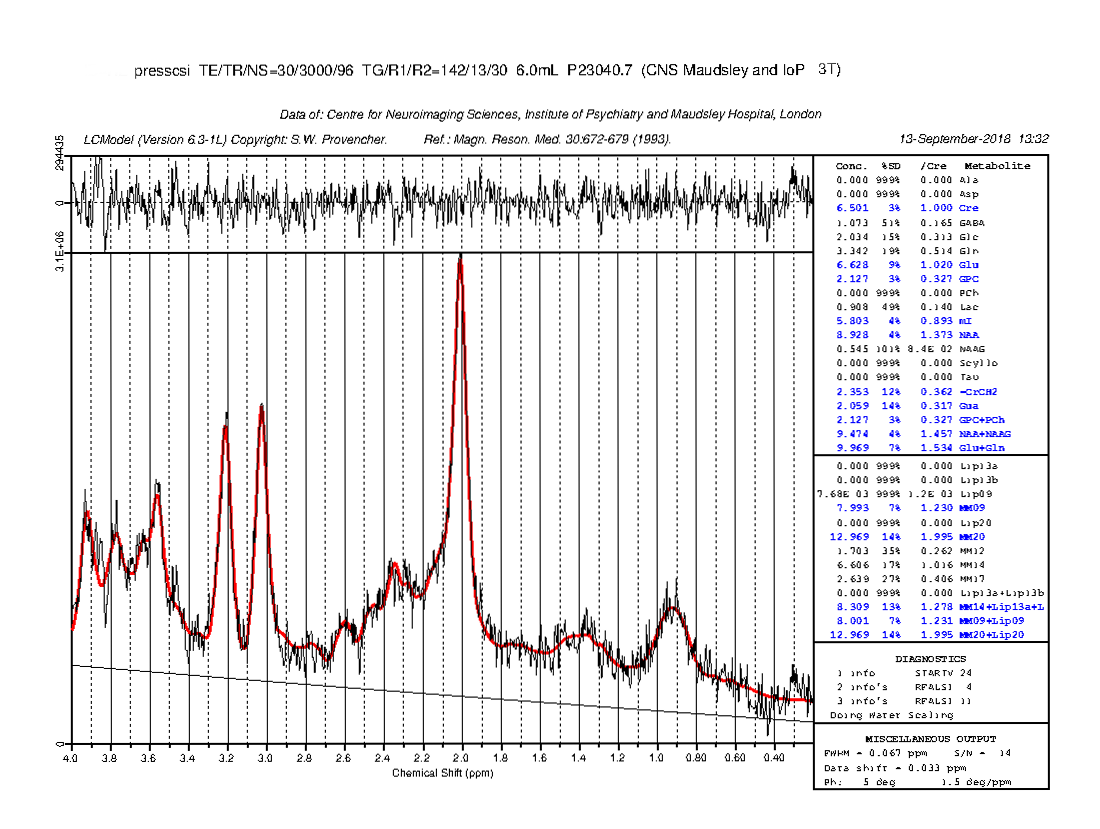


Figure S3: Example spectra obtained from one psychosis patient. Glu = glutamate; Cho = choline; Cr = creatine; Glx = glutamate + glutamine; NAA, N-acetylaspartate; ppm, parts per million.

**^1^H-MRS data quality**

Mean signal-to-noise ratios (SNR), full width at half maximum (FWHM), and Cramer-Rao lower bound (CRLB) for the participants are displayed in the table below. SNR, FWHM, and CRLB did not differ significantly within the psychosis patients across the placebo and CBD conditions. Additionally, no significant differences were observed for GM, WM, or CSF within the psychosis patients across the placebo and CBD conditions.

| **Characteristic** | **PSY-PLB** | **PSY-CBD** | ***Statistics*** | |
| --- | --- | --- | --- | --- |
|  | **Mean (SD)** | | |  |
| SNR | 13.67 (2.71) | 13.8 (4.092) | p = 0.58 | |
| FWHM | 0.069 (0.024) | 0.064 (0.023) | p = 0.15 | |
| Glu CRLB | 9.53 (2.2) | 9.47 (2.32) | p = 0.58 | |
| Glu + Gln CRLB | 9.73 (2.05) | 9.8 (2.24) | p = 0.92 | |
| GM | 0.57 (0.063) | 0.56 (0.052) | p = 0.3 | |
| WM | 0.38 (0.077) | 0.39 (0.081) | p = 0.12 | |
| CSF | 0.046 (0.029) | 0.052 (0.034) | p = 0.61 | |

SNR = signal-to-noise ratio, FWHM = full width at half maximum, Glu CRLB = Cramer-Rao lower bound for glutamate, Glu + Gln CRLB = Cramer-Rao lower bound for glutamate + glutamine, GM = grey matter, WM = white matter, CSF = cerebrospinal fluid.

# References

AGURELL, S., CARLSSON, S., LINDGREN, J. E., OHLSSON, A., GILLESPIE, H. & HOLLISTER, L. 1981. Interactions of delta 1-tetrahydrocannabinol with cannabinol and cannabidiol following oral administration in man. Assay of cannabinol and cannabidiol by mass fragmentography. *Experientia,* 37**,** 1090-2.

BERGAMASCHI, M. M., QUEIROZ, R. H., ZUARDI, A. W. & CRIPPA, J. A. 2011. Safety and side effects of cannabidiol, a Cannabis Sativa constituent. *Curr Drug Saf,* 6**,** 237-49.

BHATTACHARYYA, S., MORRISON, P. D., FUSAR-POLI, P., MARTIN-SANTOS, R., BORGWARDT, S., WINTON-BROWN, T., NOSARTI, C., CM, O. C., SEAL, M., ALLEN, P., MEHTA, M. A., STONE, J. M., TUNSTALL, N., GIAMPIETRO, V., KAPUR, S., MURRAY, R. M., ZUARDI, A. W., CRIPPA, J. A., ATAKAN, Z. & MCGUIRE, P. K. 2010. Opposite effects of delta-9-tetrahydrocannabinol and cannabidiol on human brain function and psychopathology. *Neuropsychopharmacology,* 35**,** 764-74.

FUSAR-POLI, P., ALLEN, P., BHATTACHARYYA, S., CRIPPA, J. A., MECHELLI, A., BORGWARDT, S., MARTIN-SANTOS, R., SEAL, M. L., O'CARROL, C., ATAKAN, Z., ZUARDI, A. W. & MCGUIRE, P. 2010. Modulation of effective connectivity during emotional processing by Delta 9-tetrahydrocannabinol and cannabidiol. *Int J Neuropsychopharmacol,* 13**,** 421-32.

MARTIN-SANTOS, R., CRIPPA, J. A., BATALLA, A., BHATTACHARYYA, S., ATAKAN, Z., BORGWARDT, S., ALLEN, P., SEAL, M., LANGOHR, K., FARRE, M., ZUARDI, A. W. & MCGUIRE, P. K. 2012. Acute effects of a single, oral dose of delta-9-tetrahydrocannabinol (THC) and cannabidiol (CBD) administration in healthy volunteers. *Curr Pharm Des,* 18**,** 4966-79.

SANTOS, P. M., LOPEZ-GARCIA, P., NAVARRO, J. S., FERNANDEZ, A. S., SADABA, B. & VIDAL, J. P. 2007. False positive phencyclidine results caused by venlafaxine. *Am J Psychiatry,* 164**,** 349.

SENA, S. F., KAZIMI, S. & WU, A. H. 2002. False-positive phencyclidine immunoassay results caused by venlafaxine and O-desmethylvenlafaxine. *Clin Chem,* 48**,** 676-7.
